# Supplementary material for: Evidence of a Novel Mitochondrial Signature in Systemic Sclerosis Patients with Chronic Fatigue Syndrome
Source: Int J Mol Sci. 2023 Jul 27;24(15):12057. doi: 10.3390/ijms241512057 (PMC10418481; doi:10.3390/ijms241512057)
Supplement: Supplementary file 1 [file ijms-24-12057-s001.zip › ijms-2503426-supplementary.pdf]

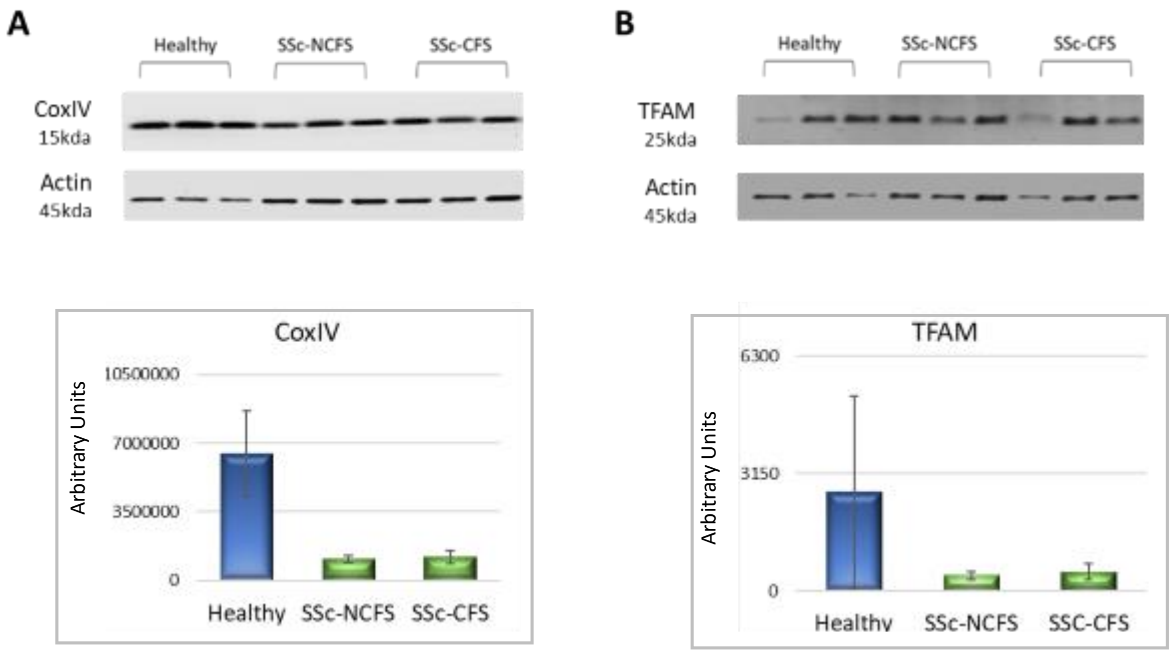

Figure S1. Representative Western blots of CoxIV and TFAM protein expression in patient and control PBMCs (three individuals in each group). Expression is represented as median ratio from 3 individuals, quantified from protein bands normalized to β-Actin. No significant difference in the expression of CoxIV and TFAM was observed between the disease groups, though both proteins appear lower in SSc patients, compared to controls.

Fatigue Related Factors

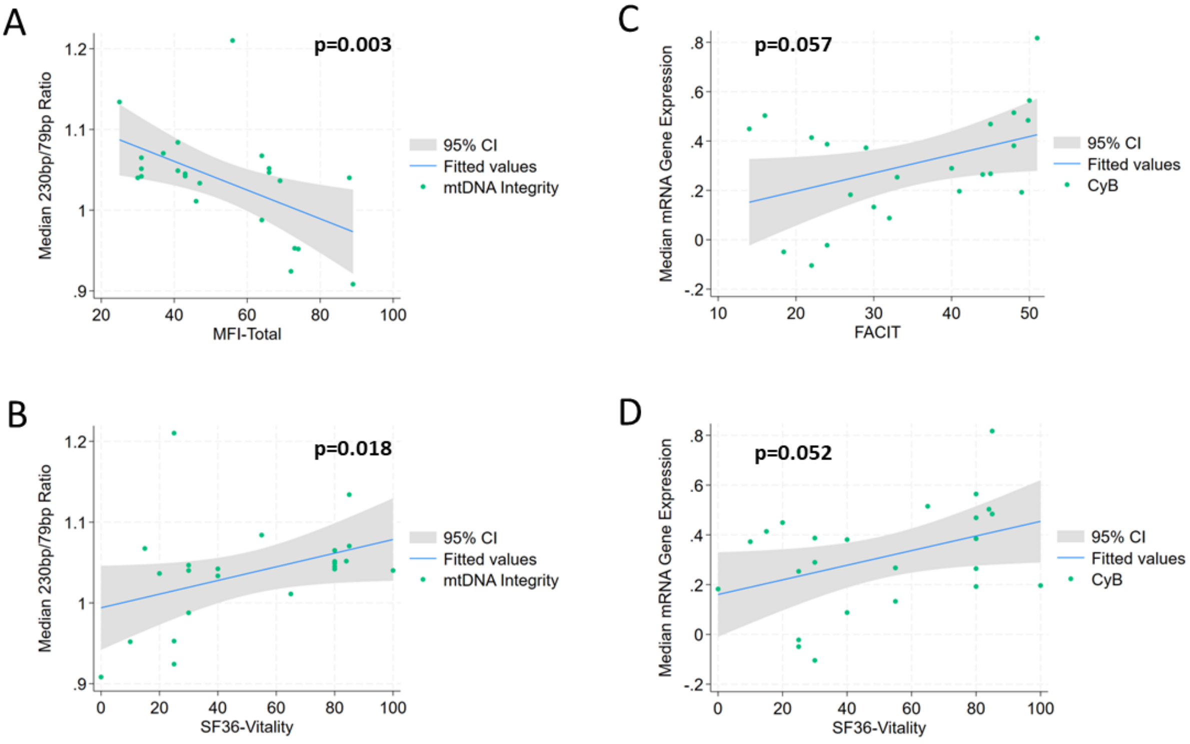

Figure S2. CyB expression had a trend towards significance with fatigue related FACIT and SF36-Vitality scores, whilst mtDNA integrity had a significant correlation with both (A-D).

Disease Related Factors

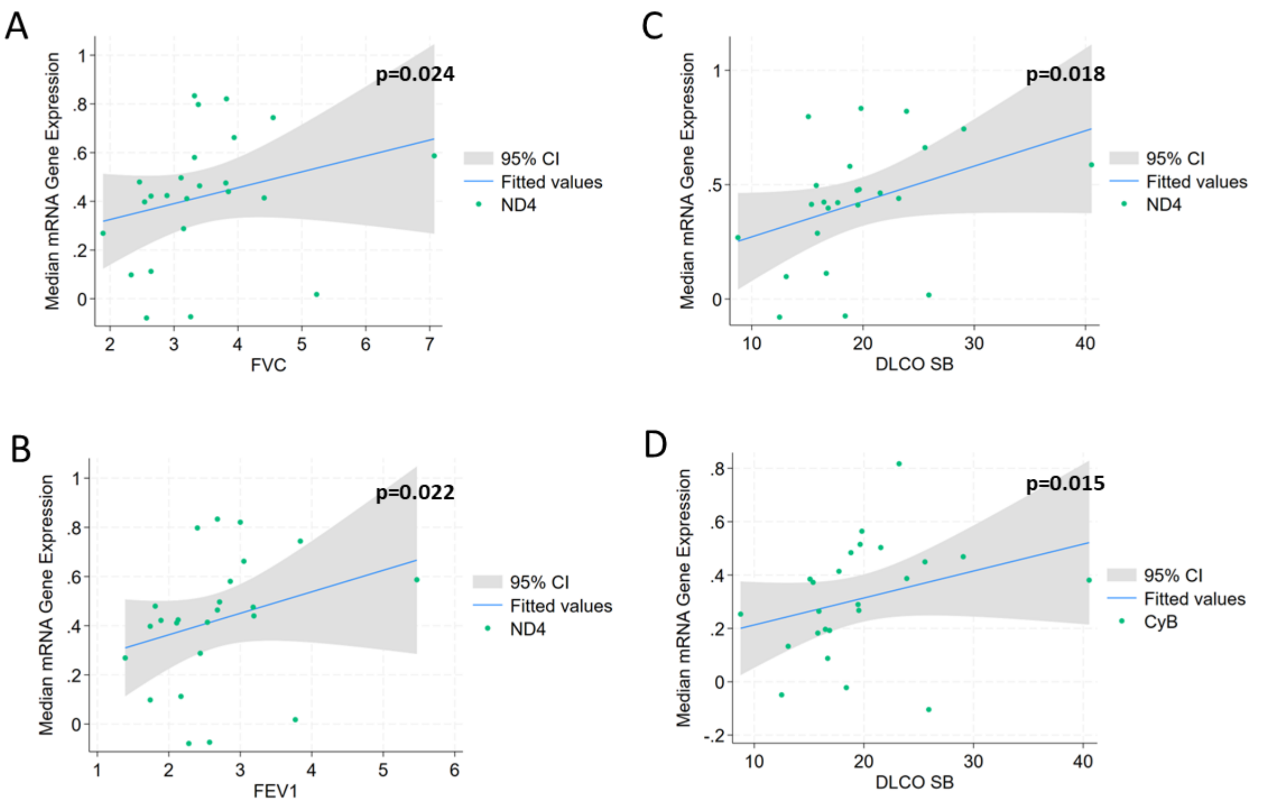

Figure S3. Correlation plots representing variables that correlated with mitochondrial measures using Spearman's rho. ND4 and CyB expression were shown to correlate with disease associated factors, FVC, FEV_1 and DLCO SB (A-D).

Table S1. Patient demographics and clinical findings. Fibromyalgia and SSc patients without ME/CFS compared to SSc patients who do present with ME/CFS.

	**SSc-CFS (n=12)**	**SSc-NCFS (n=12)**	***p***	**Healthy (n=10)**	***p***
Continuous variables	*Median (IQR)*	*Median (IQR)*		*Median (IQR*	
**Age**	47.5 (46;57)	53 (35.5;63)	0.920	46 (36;51)	0.389
**BMI**	30.4 (25.3;36.0)	27.8 (22.9;32.9)	0.178	22.5 (21.4;34.2)	0.095
**CRP**	3.5 (2.3;6.5)	2.7 (0.9;7.9)	0.579	0.6 (0.5;4.2)	0.146
**TSH**	2.63 (1.8;3.4)	1.54 (0.83;2.15)	**0.026**	1.29 (0.81;1.47)	**0.009**
**Sleep Disturbances**	13 (8;14)	3.5 (2;6.5)	**<0.001**	2.5 (1;9)	**0.001**
**SF-36**					
Physical Function	42.5 (35;55)	95 (85;100)	**<0.001**	100 (100;100)	**<0.001**
Role Physical	0.0 (0;0)	100 (100;100)	**<0.001**	100 (100;100)	**<0.001**
Bodily Pain	45 (27.5;51.2)	85 (77.5;90)	**<0.001**	100 (90;100)	**<0.001**
General Health	27.5 (20;45)	67.5 (55;80)	**<0.001**	90 (75;95)	**<0.001**
Vitality	25 (17.5;35)	80 (60;82.5)	**<0.001**	80 (70;80)	**<0.001**
Social Function	50 (50;75)	100 (87.5;100)	**<0.001**	100 (87.5;100)	**<0.001**
Role Emotion	100 (33.3;100)	100 (83.3;100)	0.277	100 (100;100)	0.060
Mental Health	64 (50;84)	86 (68;92)	0.068	88 (84;88)	**0.009**
Total	42.5 (34.1;53.8)	85.7 (77.0;92.3)	**0.001**	94.3 (90.5;95.3)	**<0.001**
**WPI**	6.5 (4.5;13)	1 (0;3)	**<0.001**	0.5 (0;2)	**<0.001**
**SSS**	7 (6.5:8)	3 (2;3)	**<0.001**	1 (1;2)	**<0.001**
**Cognitive Failure**	42.5 (18.5;51)	22 (16;31)	0.061	26.5 (12;38)	0.185
**HADS_depression**	5 (5;8.5)	1.5 (0.5;4)	**0.002**	0.5 (0;5)	**<0.001**
**HADS_anxiety**	8 (5.5;12)	4 (2;7)	**0.012**	4 (2;5)	**0.006**
**MFI**					
General fatigue	18 (15;18.5)	9 (8.5;10)	**<0.001**	5.5 (5;8)	**<0.001**
Physical fatigue	13.5 (12.5;17)	6 (5;10)	**<0.001**	5.5 (4;6)	**<0.001**
Mental fatigue	12.5 (7.5;16)	7.5 (6;9)	**0.039**	5.5 (4;8)	**<0.001**

Reduced activity	12.5 (10.5;14)	5.5 (4;9.5)	**<0.001**	5 (4;8)	**<0.001**
Reduced motivation	11.5 (10;13)	6.5 (5;10)	**0.003**	6 (5;7)	**<0.001**
Total	67.5 (60;73.5.)	39 (31;43)	**<0.001**	28.5 (26;34)	**<0.001**
**FACIT fatigue**	24 (20.2;29.5)	48 (44;49.8)	**<0.001**	50 (50;52)	**<0.001**

Abbreviations: SSc-CFS :early systemic sclerosis with ME/CFS; SSc-NCFS: early scleroderma without ME/CFS; FM: Fibromyalgia; IQR: interquartile range; BMI: body mass index; CRP: C reactive protein; TSH: Thyroid stimulating hormone; ILD: interstitial lung disease ME/CFS: myalgic encephalomyelitis/chronic fatigue syndrome; SF-36: 36-item short form health survey; HADS: Hospital anxiety and depression scale; MFI: multidimentional fatigue inventory; FACIT: functional assessment of chronic illness therapy; p: p value.

Table S2. Immunomodulatory and vasodilator medication use and clinical findings.

	**No Immunomodulator or Vasodilator**	**Immunomodulator**	**Vasodilator**	**Immunomodulator and Vasodilator**	**p-value**
	Yes (%)	*Yes (%)*	*Yes (%)*	*Yes (%)*	
**ILD**	0/3 (0.0)	1/5 (20.0)	1/8 (12.5)	2/8 (25.0)	0.767
**Digital ulcers**	0/3 (0.0)	0/5 (0.0)	5/8 (62.5)	1/8 (12.5)	**0.025**
**Arthralgia**	0/3 (0.0)	2/5 (40.0)	0/8 (0.0)	7/8 (87.5)	**0.002**
**GERD**	0/3 (0.0)	1/5 (20.0)	3/8 (37.5)	3/8 (37.5)	0.577
**Sleep Disturbances**	2/3 (66.6)	3/5 (60.0)	3/8 (37.5)	5/7 (62.5)	0.581
**FM**	1/3 (33.3)	2/5 (40.0)	2/8 (25.0)	3/8 (37.5)	0.938
**HADS depression**	1/3 (33.3)	1/5 (20.0)	0/8 (0.0)	0/8 (0.0)	0.187
**HADS anxiety**	1/3 (33.3)	1/5 (20.0)	1/8 (12.5)	1/8 (12.5)	0.840
**ME/CFS**	1/3 (33.3)	3/5 (60.0)	3/8 (37.5)	5/8 (62.5)	0.675

Table S3. Spearman correlations comparing correlates of ETC gene expression.

	**ND4**	**CyB**	**Cox7C**	**mtDNA Integrity**
	**rho**	**rho**	**rho**	**rho**
**Age**	-0.35	-0.31	0.22	0.11
**CRP**	-0.32	-0.16	0.04	-0.16
**BMI**	-0.13	-0.29	0.34	-0.37
**TSH**	0.14	-0.04	-0.05	-0.04
**mRSS**	-0.41*	-0.45*	-0.09	-0.03
**DLCO SB**	0.48*	0.49*	-0.01	0.16
**FVC**	0.46*	0.33	-0.19	0.02
**FEV_1**	0.46*	0.28	-0.23	-0.06
**PSQI**	-0.34	-0.41^+	0.34	-0.38
**WPI**	-0.20	-0.25	0.21	-0.42*
**SSS**	-0.31	-0.33	0.29	-0.29
**CFQ**	-0.04	-0.16	0.24	-0.35
**HADS-Anxiety**	-0.30	-0.31	0.29	-0.36
**HADS-Depression**	-0.31	-0.40^+	0.17	-0.30
**FACIT**	0.30	0.40^+	-0.46*	0.34
**MFI**	-0.27	-0.37	0.29	-0.59**
**SF-36 Vitality**	0.23	0.40^+	-0.18	0.49*

+ = 0.05; * = <0.05; ** = <0.01.

Table S4. Mitogen^{Dx} antibody panel.

	**Systemic Sclerosis Panel**
**Antibodies** (MitogenDx laboratories, Calgary, Alberta)	Anti-CENP A + B, Topo-I/Scl-70, RNA polymerase III, fibrillarin, Th/To/hPOP1, Ku, PDGFR, Ro52/TRIM21, PM21, PM/Scl-75, PM/Scl-100, Nor90/Hubf, Jo-1, Mi2, Mi2-α, Mi2β, MDA5, NXP2, TIF1γ, PL7, PL12, SRP, EJ, OJ

Questionnaires

All questionnaires listed below, are validated, reliable tools, which are freely available for use.

All patients completed the DePaul Questionnaires (DSQ-2), a self-reported validated measure to assess the presence of ME/CFS symptoms such as fatigue, post-exertional malaise, sleep, pain, and neurological/cognitive impairments, autonomic, neuroendocrine, and immune-related symptoms. DePaul questionnaire contains questions with the purpose of determining the frequency and severity of ME/CFS symptoms. Frequency is rated on a scale of 0 to 4, with 0=none of the time, 1= little of the time, 2= about half of the time, 3= most of the time, 4= all of the time over the past 6 months; and severity rated again on a scale of 0 to 4, with 0= no symptoms, 1= mild, 2= moderate, 3=severe and 4 very severe. The patients considered as having ME/CFS needed to meet ≥ 5 out of the 6 diagnostic criteria [1].

The MFI is a self-reported 20-item scale designed to evaluate five dimensions of fatigue: general fatigue, physical fatigue, reduced motivation, reduced activity, and mental fatigue. Items are scored 1–5, with 10 [fatigue-] positively phrased items reverse scored (items 2, 5, 9, 10, 13, 14, 16, 17, 18, 19). In the final score, higher scores represent more acute levels of fatigue [2].

The Functional Assessment of Chronic Illness Therapy (FACIT), a self-reported 13-item questionnaire that measures the level of fatigue during usual daily activities over the past 7 days. Items are measure on a four-point Likert scale (0= not at all, 1= a little bit, 2= somewhat, 3= quite a bit and 4= very much) except items 7 and 8 which are reversed scored. Score ranges between 0-52 with a score less than 30 indicates serve fatigue and the higher the score, the better the quality of life [3].

For assessing self-reported health-related quality of life in patients, we used The Short Form (36) Health Survey questionnaire, with scores ranging from 0-to 100. Lower scores in the following categories: vitality, physical functioning, body pain, the general perception of health, physical role functioning, emotional role functioning, social role functioning, and mental health, indicated more fatigue and disability. These eight domains can be summarized into a Physical Component Score (PCS) and a Mental Component Score (MCS). A final score for each domain is provided between 0-100, with 0 being the worst possible health and 100 the best possible health. The score is standardized against normative population data, where the mean score ±SD of 50 ± 10 is the population normative score [4].

For the assessment of FM, The American College of Rheumatology Preliminary Diagnostic Criteria for Fibromyalgia and Measurement of Symptoms Severity (2010) was used. This self-reported tool evaluates the widespread pain index (WPI) and the symptoms severity of score (SSS), combined with multiple somatic symptoms. A patient meets the diagnostic criteria for fibromyalgia if the following 3 conditions are met: The WPI score is greater than or equal to 7 AND the SS score is greater than or equal to 5 or the WPI score is from 3 to 6 AND the Score is greater than or equal to 9. Symptoms have been present at a similar level for at least 3 months [5, 6].

The Hospital Anxiety and Depression Scale (HADS) questionnaire, is a 14-item self-reported screening scale to identify the presence of anxiety and depression in patients was used in our survey. This tool contains 7 anxiety-related and 7 depression-related items, with scores of each ranging from 0-3, and with a total score of 11 or above indicative of abnormal results [7].

Sleep disturbances were assessed using The Pittsburgh Sleep Quality Index (PSQI), which is a self-reported questionnaire to evaluate a wide variety of factors relating to sleep quality. These include estimates of sleep duration, latency, frequency, and severity of specific sleep-related problems. This tool measure seven domains (subjective sleep quality, sleep latency, sleep duration, habitual sleep efficiency, sleep disturbances, use of sleep medication, and daytime dysfunction) over the past month, with answers based on a 0-3 (0= not during the past month, 1= less than once a week, 2= once or twice a week and 3= three or more times week) scale. A global score of 5 or greater indicates poorer sleep quality [8].

The Cognitive Failure questionnaire consists of 25 self-reported items to analyze the frequency of cognitive failure. Factors assessed included absent-

mindedness, slips and errors, memory, and motor functioning in everyday life. The scoring is from 0-100, with scores of ≥43 is indicative of cognitive impairment [9].

Table S4. Questionnaires used for assessing patient symptoms.

Abbreviation	Questionnaire	Reference
DSQ-2	DePaul Symptom Questionnaire	[1]
MFI	Multidimensional Fatigue Inventory	[2]
FACIT	The Functional Assessment of Chronic Illness Therapy – Fatigue	[3]
SF36	Short Form - 36	[4]
WPI	Widespread Pain Index	[5, 6]
SSS	Symptom Severity Scale	[5, 6]
HADS Anxiety	Hospital Anxiety and Depression Scale	[7]
HADS Depression	Hospital Anxiety and Depression Scale	[7]
PSQI	Pittsburgh Sleep Quality Index	[8]
CFQ	Cognitive Failure Questionnaire	[9]

Table S5. Primers sequences for mtDNA integrity assay.

Primer	Sequence
GAPDH F	CCCCACACACATGCACTTACC
GAPDH R	CCTAGTCCCAGGGCTTTGATT
203F	CAGCCGCTATTAAAGGTTCG
203R	GGGCTCTGCCATCTTAACAA
79F	CAGCCGCTATTAAAGGTTCG
79R	CCTGGATTACTCCGGTCTGA

Western Blot Analysis

Total protein extracts from were prepared from PBMCs in RIPA lysis buffer, containing 1X protease (Halt™ Phosphatase inhibitor cocktail (78429, Thermo Scientific)), phosphatase inhibitors (Halt™ Phosphatase inhibitor cocktail (78420, Thermo Scientific)) and Phenylmethanesulfonyl fluoride (P7626, Sigma-Aldrich). Protein concentrations were measured using the Micro BCA™ Protein Assay Kit (Thermo Fisher). Fifty microgram protein extracts were resolved on 12% SDS polyacrylamide gels. Proteins were transferred onto a polyvinylidene fluoride membrane using a BioRad gel blotting apparatus. Membranes were incubated with a primary antibody against CoxIV (33985, Abcam), TFAM (8076, Cell Signaling) and B-Actin (8457, Cell Signaling) (1/1000 in diluent) overnight at 4 C and with secondary antibodies (800CW goat anti rabbit. 926-32211, IRDye and 680RD goat anti mouse, 926-68070, LI-COR Biosciences) (1/15000 in diluent) for 60 min at room temperature. Bands were normalized to β-Actin loading control. Western blot bands were quantitated using Image studio Lite software (LI-COR Biosciences).

References

1. Bedree, H.; Sunnquist, M.; Jason, L.A. The DePaul Symptom Questionnaire-2: a validation study. *Fatigue: Biomed. Heal. Behav.* **2019**, *7*, 166–179, https://doi.org/10.1080/21641846.2019.1653471.
2. Smets, E.M.A.; Garssen, B.; Bonke, B.; De Haes, J.C.J.M. The multidimensional fatigue inventory (MFI) psychometric qualities of an instrument to assess fatigue. *J. Psychosom. Res.* **1995**, *39*, 315–325.
3. Yellen, S.B.; Cella, D.F.; Webster, K.; Blendowski, C.; Kaplan, E. Measuring fatigue and other anemia-related symptoms with the Functional Assessment of Cancer Therapy (FACT) measurement system. *J. Pain Symptom Manag.* **1997**, *13*, 63–74, https://doi.org/10.1016/s0885-3924(96)00274-6.
4. Ware, J.E., Jr.; Sherbourne, C.D. The MOS 36-item short-form health survey (SF-36). I. Conceptual framework and item selection. *Med. Care* **1992**, *30*, 473–483.
5. Wolfe, F., et al., 2016 Revisions to the 2010/2011 fibromyalgia diagnostic criteria. Semin Arthritis Rheum, 2016. 46(3): p. 319-329.
6. Wolfe, F., et al., The American College of Rheumatology preliminary diagnostic criteria for fibromyalgia and meas-ure-ment of symptom severity. Arthritis Care Res (Hoboken), 2010. 62(5): p. 600-10.
7. Herrmann, C. International experiences with the Hospital Anxiety and Depression Scale-a review of validation data and clinical results. *J. Psychosom. Res.* **1997**, *42*, 17–41

8. Buysse, D.J.; Reynolds, C.F., III; Monk, T.H.; Berman, S.R.; Kupfer, D.J. The Pittsburgh sleep quality index: A new instrument for psychiatric practice and research. *Psychiatry Res.* **1989**, *28*, 193–213, doi:10.1016/0165-1781(89)90047-4.
9. Broadbent, D.E.; Cooper, P.F.; FitzGerald, P.; Parkes, K.R. The Cognitive Failures Questionnaire (CFQ) and its correlates. *Br. J. Clin. Psychol.* **1982**, *21*, 1–16, https://doi.org/10.1111/j.2044-8260.1982.tb01421.x.
